# A case of congenital combined pituitary hormone deficiency who showed respiratory distress and hypoglycemia soon after birth

**DOI:** 10.1186/1687-9856-2015-S1-P116

**Published:** 2015-04-28

**Authors:** Satomi Koyama, Shinichiro Ariga, Katsura Kariya, Yayoi Tsuboi, Hiroshi Suzumura, Osamu Arisaka

**Affiliations:** 1 Department of Pediatrics, Dokkyo Medical University, Mibu, Tochigi, Japan

## 

Congenital combined pituitary hormone deficiency (CCPHD) is a rare disease, presenting with respiratory distress, hypoglycemia and jaundice soon after birth. It is caused by .pituitary aplasia or hypoplasia or abnormality of transcription factor which related to pituitary development. We describe a Japanese patient with CCPHD, who presented with respiratory distress, hypoglycemia and jaundice soon after birth.

The patient is now 13 months old Japanese girl. She was born by cesarean section because of fetal distress with 39 weeks and 4 days gestation after an uneventful pregnancy. Her parents were unrelated. Birth length and weight were 43.8cm (-2.2SD) and 2,100g and Apgar scores were 8 at both of 1 and 5 min. She had showed tachypnea and cyanosis since soon after birth and taken nasal-CPAP. However respiratory distress had not improved. She had also showed jaundice and hypoglycemia and was treated with phototherapy and infusion therapy. At 6 days old, we found her FT4 level was 0.4 pg/ml and TSH level was below 0.01 μIU/ml. She was suspected CPHD and anterior pituitary hormone levels were assessed. All of anterior pituitary hormones were undetectable. Anterior pituitary was not visible, posterior pituitary was detected at normal position and there were no other abnormality on brain magnetic resonance imaging. We diagnosed her as CCPHD and hydrocortisone was started since 6 days old and levothyroxine since 8 days old. After that respiratory distress and jaundice had improved rapidly. But hypoglycemia had appeared again after interrupting infusion therapy. GH level after arginine stimulation was also undetectable. She had also started growth hormone therapy since 25 days old and she has had no hypoglycemic episodes after that. Her length and weight were 70.3cm (-1.2SD) and 8,485g at 1 year old. GH replacement therapy that was started soon after diagnosis was able to prevent the severe growth retardation. She has no gene abnormalities of transcription factors, HESX1, LHX4 and OTX2, that involves pituitary development. Further studies are needed for investigating genetic basis of this patient.

Careful and prompt clinical, endocrinological and neuroradiological assessment are needed for diagnosis of hypopituitarism in patients who show prolonged respiratory distress, hypoglycemia or jaundice soon after birth.

Written informed consent was obtained from the patient's parent or guardian for publication of this abstract and any accompanying images. A copy of the written consent is available for review by the Editor of this journal.

